# Silently Wrapped: Embolization and Surgical Strategy for Giant Sciatic-Sparing Myxoid Liposarcoma—Case Report

**DOI:** 10.3390/reports8030124

**Published:** 2025-07-28

**Authors:** Radu Aurelian Vișan, Victor Baluța

**Affiliations:** Department of Orthopedics and Traumatology, “Carol Davila” University of Medicine and Pharmacy, 020021 Bucharest, Romania

**Keywords:** myxoid liposarcoma, sciatic nerve, embolization, case report

## Abstract

**Background and Clinical Significance:** Myxoid liposarcoma (MLS) is a malignant soft tissue tumor that often presents as a painless, slow-growing mass and is known for its atypical extrapulmonary metastatic pattern. Although sciatic nerve involvement is rare, when present, it usually causes neurologic symptoms. In this case, a large MLS silently expanded and completely encased the sciatic nerve without causing deficits, highlighting the importance of early imaging, multidisciplinary planning, and individualized surgical strategy in managing complex soft tissue sarcomas. **Case Presentation:** This case report describes a 67-year-old male with a 30 cm encapsulated myxoid liposarcoma of the posterior left thigh. The tumor had grown insidiously over one year and completely encased the sciatic nerve without causing pain, paresthesia, or motor impairment. Selective embolization was performed preoperatively to minimize blood loss. A posteromedial surgical approach allowed for en bloc resection with negative margins and preservation of sciatic nerve integrity. Histopathology confirmed a myxoid liposarcoma composed primarily of spindle-shaped tumor cells. The patient experienced no postoperative complications or neurologic deficits. At the two-year follow-up, he remains disease-free with full functional recovery. **Conclusions:** This case illustrates the potential for large, asymptomatic myxoid liposarcomas to encase critical neurovascular structures without infiltration. Preoperative embolization as part of a multidisciplinary plan was key to achieving safe resection and excellent functional outcomes.

## 1. Introduction and Clinical Significance

Myxoid liposarcoma (MLS) is a malignant soft tissue tumor and one of the most common subtypes of liposarcoma, accounting for approximately 30–40% of all cases [1]. Typically, MLS develops in the soft tissues of the extremities, particularly the thigh, and is commonly found in adults in their fifth to sixth decades of life [2]. Histologically, MLS is characterized by a myxoid matrix, an arborizing capillary network (chicken-wire), and scattered lipoblasts [3]. Some cases also present with round cell components, which are associated with a more aggressive behavior [4].

A definitive diagnosis of liposarcoma is confirmed only by the histopathological result, but imaging investigations are essential for initial characterization, staging, and surgical planning. MRI is the preferred first-line investigation due to its excellent soft tissue contrast and ability to define tumor extent [5]. CT angiography (CTA) provides additional information by identifying the tumor’s vascular supply and dominant feeding arteries [6]. In selected cases, especially in large tumors where preoperative embolization is considered, MRI and CTA play complementary roles in safely planning the procedure.

Although MLS is considered a low- to intermediate-grade sarcoma, it has a unique metastatic pattern. Other liposarcomas metastasize primarily to the lungs, whereas MLS tends to metastasize to extrapulmonary sites such as the retroperitoneum, bones, or soft tissues [7]. Standard treatment involves wide surgical excision, often combined with radiotherapy or, in selected cases, chemotherapy [8]. Preoperative planning is essential, especially in cases of large tumors adjacent to major neurovascular structures, because preserving function is a priority.

This case report aims to describe a rare presentation of a giant myxoid liposarcoma of the posterior left thigh that silently grew around the sciatic nerve without causing any neurological symptoms, and was successfully treated through selective arterial embolization followed by nerve-sparing surgery. Its uniqueness lies in the tumor’s silent progression, and the management of the case highlights the importance of individualized preoperative planning and a multidisciplinary approach in large-volume soft tissue sarcomas.

## 2. Case Presentation 

A 67-year-old male presented to the orthopedic emergency department with pain and swelling in the left thigh following a direct blunt trauma sustained earlier that day. The patient reported a slowly growing mass in the same region over the last year, which he had neglected. On clinical examination, the left thigh was significantly enlarged on the posteromedial side, with diffuse swelling, ecchymosis and tenderness to palpation. Passive and active range of motion at the hip and knee were preserved, but the patient complained of pain while weight-bearing. There were no signs of motor or sensory deficits, and specifically, no symptoms suggestive of sciatic nerve involvement such as radiating pain, paresthesia, or muscle weakness. The patient has a known history of arterial hypertension, supraventricular tachycardia, and chronic bronchitis.

Initial imaging included plain radiographs of the left thigh and chest. The upper-leg X-ray revealed posteromedial soft tissue expansion without any evidence of osseous involvement or pathological fracture. The chest radiograph showed no pulmonary lesions. The patient was treated conservatively with NSAIDs, analgesics and the RICE protocol for the traumatic component, and an MRI of the thigh was scheduled to evaluate the underlying mass.

**Figure 1:**
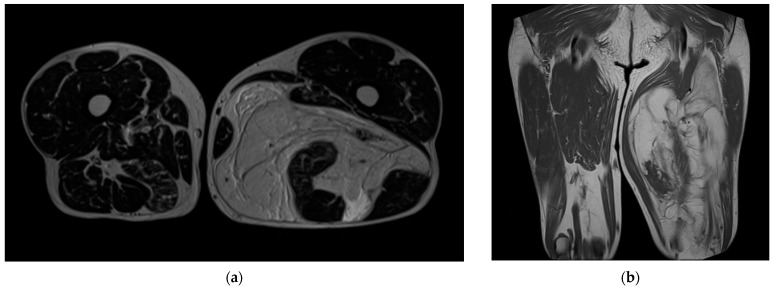
MRI images of the left thigh: (**a**) axial view; (**b**) coronal view.

The MRI of the left thigh (Figure 1) revealed a large, well-demarcated soft tissue tumor located in the posterior compartment, at the mid-thigh level. The mass measured approximately 30 cm in its longest axis, appeared multilobulated with internal septations, and showed a predominantly hyperintense signal on T2-weighted MRI sequences, along with areas of contrast enhancement.

It was in close proximity to the sciatic nerve, which appeared to be encased, but no displacement or compression were noted. There were no signs of invasion into adjacent bone or vascular structures. Based on imaging characteristics, a soft tissue sarcoma was suspected. The patient underwent a contrast-enhanced CT scan of the chest, abdomen, and pelvis, which showed no evidence of secondary lesions.

Ten days later, an ultrasound-guided percutaneous core needle biopsy of the mass was performed. Cytopathological examination revealed the presence of discohesive lipoblast-like cells and scattered erythrocytes. The findings raised suspicion for a round cell sarcoma. The case was discussed in a multidisciplinary tumor board, and the decision was to proceed with surgical excision.

**Figure 2:**
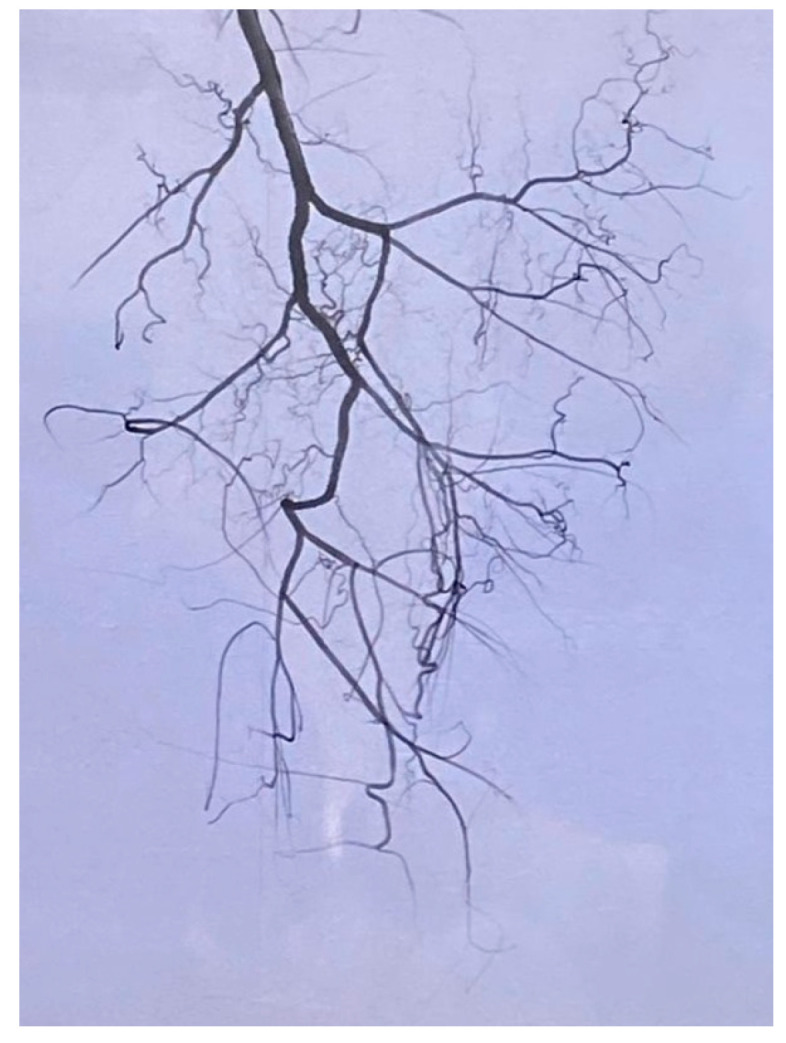
Angiographic image during embolization, showing an enlarged deep femoral artery and neoformed vessels.

Given the tumor’s considerable size and localization, a preoperative angiographic evaluation was performed to assess its vascular supply. The angiography revealed an enlarged left deep femoral artery along with its hypertrophied branches (Figure 2). Peripheral neoformed vessels supplying the tumor were also visualized. Embolization was performed selectively on these muscular branches using both non-resorbable particles (Embozene^®^) and resorbable agents (Gelaspon^®^). The procedure was well-tolerated, with no complications, and surgical resection was carried out the following day.

Surgical excision was performed through a posteromedial approach centered on the tumor mass. Intraoperatively, an encapsulated mass with a multilobulated and lipomatous appearance was discovered. The tumor showed no adherence to the surrounding muscle layers, and through careful dissection along the natural cleavage planes, it was successfully mobilized. It occupied a large portion of the posterior thigh compartment and was found to surround the sciatic nerve circumferentially, without infiltrating it. The nerve was meticulously dissected and preserved throughout the procedure. Blood loss was significantly reduced by the prior embolization, and the mass was excised en bloc with wide margins. The tumor measured approximately 30 cm in diameter and weighed 2.5 kg.

**Figure 3:**
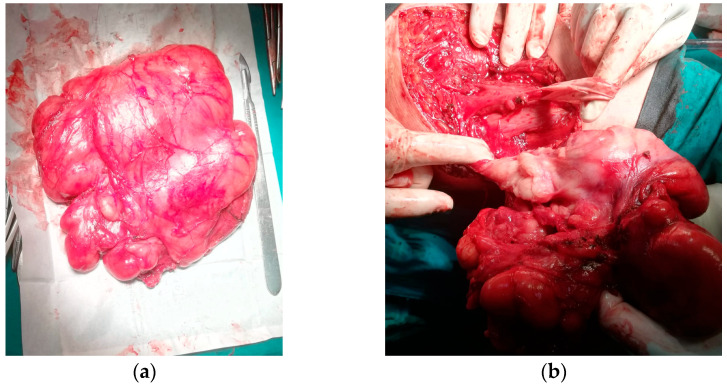
Intraoperative images. (**a**) The entire encapsulated tumor after resection. (**b**) The sciatic nerve dissected and preserved.

**Figure 4:**
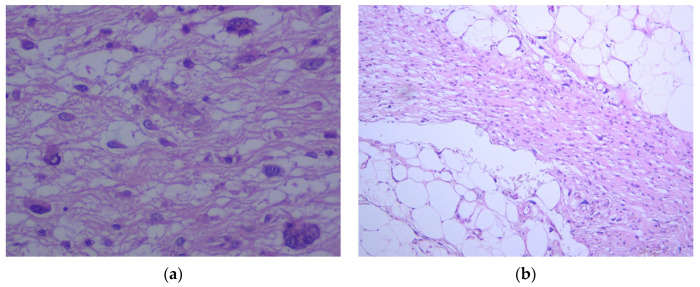
Histopathological features of the resected tumor. (**a**) HE, 40×, showing lipoblasts and multinucleated tumor cells embedded in a myxoid stroma. (**b**) HE, 10×, showing spindle-shaped tumor cells with nuclear atypia.

Gross examination revealed a well-encapsulated, multilobulated tumor with a yellowish, greasy, lipoma-like cut surface (Figure 3). Histologically, the mass was composed of adipocyte cells with nuclear atypia (lipoblasts) in a loose myxoid stroma. The background featured characteristic arborizing, thin-walled capillaries arranged in a “chicken-wire” pattern, along with areas of stromal fibrosis. Scattered spindle-shaped cells with nuclear atypia were also observed, some of which were multinucleated and had abundant eosinophilic cytoplasm (Figure 4). The surgical margins were clear. The histopathological diagnosis was myxoid liposarcoma composed primarily of spindle-shaped tumor cells. Immunohistochemical staining was performed and supported the diagnosis. DDIT3 showed strong nuclear positivity, while S100 was focally positive. MDM2, CD34, and Desmin were negative. Based on the American Joint Committee on Cancer (AJCC) staging system for soft tissue sarcomas, the tumor corresponded to stage IIIB (T4, N0, M0).

No local or systemic postoperative complications were observed. The patient began early active and passive mobilization under supervision. As in the preoperative period, no neurological deficits or complaints were noted. He was referred to an oncology center for continued follow-up and was subsequently monitored every six months with imaging studies, without receiving any adjuvant oncologic therapy. At both the one-year and two-year follow-up visits in the orthopedic department, the patient remained in good general condition, with no clinical or imaging evidence of local recurrence or distant metastasis.

## 3. Discussion

Myxoid liposarcoma (MLS) is a distinct type of liposarcoma, typically found in the limbs and characterized by a myxoid stroma, arborizing capillaries, and lipoblasts [3]. Sometimes it presents as a benign lesion due to slow growth and minimal symptoms [1].

One particularly interesting aspect of this case is how closely the tumor was related to the sciatic nerve—it surrounded the nerve but did not invade it. Despite this close contact, the patient had no neurological symptoms before or after surgery. While sciatic nerve involvement can occur in liposarcomas, especially in more aggressive types, it usually causes pain, paresthesia, or muscle weakness [9]. In this case, the lack of symptoms highlights how important preoperative imaging is in evaluating nerve involvement and planning a careful dissection.

This clinical presentation raises the question of how frequently such extensive sciatic involvement occurs and what surgical outcomes have been reported in similar cases. Sciatic nerve involvement in large myxoid liposarcomas is uncommon, but when present, it poses significant surgical challenges. A review by Williams et al. reported that approximately 15% of deep-seated lower-limb sarcomas involve or encase the sciatic nerve, and in selected cases, limb-sparing resections with nerve preservation can be performed successfully, achieving both oncologic control and satisfactory functional outcomes [9]. Our case aligns with this limited but growing body of evidence, demonstrating that even tumors encasing critical structures like the sciatic nerve can be safely managed without neurological compromise.

In this case, embolization before surgery made a significant difference in how the procedure was planned and carried out. Although not routinely used in soft tissue sarcoma management, preoperative embolization has shown promising results in selected cases. In a prospective series of 31 patients undergoing embolization for hypervascular bone and soft tissue tumors, 71% required no blood transfusions during surgery [10]. Similarly, a recent retrospective study found that embolization prior to sarcoma resection was associated with significantly reduced intraoperative blood loss and shorter hospital stays, with no increase in complications [11]. A comparable case was reported by Hansch, A et al., involving a hemorrhagic sarcoma that was successfully managed with transarterial embolization prior to surgery [12]. These findings support the rationale for selective arterial embolization in large-volume or hypervascular sarcomas, especially when major neurovascular structures are involved, as in this case. Angiography revealed hypertrophied branches of the deep femoral artery and peripheral neovessels supplying the mass. Embolization with both resorbable and non-resorbable agents led to an excellent outcome.

Although radiotherapy does not directly reduce the risk of intraoperative bleeding, it can contribute to tumor shrinkage, thereby facilitating surgical resection—especially in large soft tissue sarcomas or in cases where embolization is not possible. In his review, Rick L.M. Haas emphasized the effectiveness of radiotherapy as part of a multimodal strategy for soft tissue sarcomas, highlighting its role in improving local control and preserving function [13].

Beyond locoregional strategies such as embolization, systemic neoadjuvant therapies are increasingly considered in the management of large or marginally resectable soft tissue sarcomas.

Trabectedin is gaining attention as a neoadjuvant treatment for myxoid liposarcoma, especially because it directly targets the FUS-DDIT3 fusion gene typical of this tumor. Results presented at the ASCO 2024 congress showed that trabectedin can be just as effective as the traditional anthracycline–ifosfamide regimens, with the added benefit of being better tolerated—making it a valuable option in selected cases [14].

Lastly, this case emphasizes the importance of a multidisciplinary team and thorough preoperative planning. Despite the tumor’s size and silent progression over a year, wide resection with negative margins and functional preservation was achieved. At the two-year follow-up, the patient remains disease-free, indicating a successful oncological and functional outcome.

## 4. Conclusions

This report presents a rare case of a large, asymptomatic myxoid liposarcoma closely surrounding the sciatic nerve, successfully managed with a combination of selective embolization and surgical excision. Such a presentation—complete sciatic encasement without neurologic symptoms—is uncommon and underscores the variable clinical behavior of myxoid liposarcoma.

## Data Availability

The original contributions presented in the study are included in the article material, and further inquiries can be directed to the corresponding author.
